# Thymidylate synthase, dihydropyrimidine dehydrogenase, ERCC1, and thymidine phosphorylase gene expression in primary and metastatic gastrointestinal adenocarcinoma tissue in patients treated on a phase I trial of oxaliplatin and capecitabine

**DOI:** 10.1186/1471-2407-8-386

**Published:** 2008-12-23

**Authors:** Kazumi Uchida, Peter V Danenberg, Kathleen D Danenberg, Jean L Grem

**Affiliations:** 1 USC Norris Comprehensive Cancer Center, Room 5318, 1441 Eastlake Ave, Los Angeles, CA 90033, USA; 2 Response Genetics, Inc, No 620, 1640 Marengo St, Los Angeles, CA 91001, USA; 3 University of Nebraska Medical Center, 987680 Nebraska Medical Center, Omaha, NE 68198, USA

## Abstract

**Background:**

Over-expression of thymidylate synthase (TS) and dihydropyrimidine dehydrogenase (DPD) in tumor tissue is associated with insensitivity to 5-fluorouracil (5-FU). Over-expression of ERCC1 correlates with insensitivity to oxaliplatin (OX) therapy, while high thymidine phosphorylase (TP) levels predict for increased sensitivity to capecitabine (Xel).

**Methods:**

Biopsies of metastatic tumor were taken before OX (130 mg/m^2 ^day 1) given with Xel (1200–3000 mg/m^2 ^in two divided doses days 1–5 and 8–12) every 3-weeks. Micro-dissected metastatic and primary tumors were analyzed for relative gene expression by real-time quantitative polymerase chain reaction. The clinical protocol prospectively identified the molecular targets of interest that would be tested. Endpoints for the molecular analyses were correlation of median, first and third quartiles for relative gene expression of each target with response, time to treatment failure (TTF), and survival.

**Results:**

Among 91 patients participating in this trial; 97% had colorectal cancer. The median number of prior chemotherapy regimens was 2, and most had prior 5-FU and irinotecan. In paired samples, median mRNA levels were significantly higher in metastatic versus primary tumor (-fold): TS (1.9), DPD (3.8), ERCC1 (2.1) and TP (1.6). A strong positive correlation was noted between DPD and TP mRNA levels in both primary (r = 0.693, p < 0.0005) and metastatic tissue (r = 0.697, p < 0.00001). There was an association between TS gene expression and responsive and stable disease: patients whose intratumoral TS mRNA levels were above the median value had significantly greater risk of early disease progression (43% vs 17%), but this did not translate into a significant difference in TTF. ERCC1 gene expression above the third quartile was associated with a shorter TTF (median 85 vs 162 days, p = 0.046). Patients whose TS mRNA levels in metastatic tumor tissue were below the median had a longer overall survival (median 417 vs 294 days, p = 0.042).

**Conclusion:**

Target gene expression in primary tumor was significantly lower than that in paired metastatic tissue. High ERCC1 mRNA levels in metastatic tumor was associated with a shorter TTF. Lower expression of TS mRNA correlated with a lower chance of early PD with XelOX therapy and improved overall survival.

## Background

It is estimated that about 153,000 new cases of colorectal cancer will be diagnosed in the U.S. in 2007 [1]. 5-fluorouracil (5-FU), floxuridine (FUDR), capecitabine (Xel), irinotecan, oxaliplatin (OX), cetuximab, panitumumab and bevacizumab are licensed in the U.S. for use in patients with colorectal cancer. Although each of these agents has clinical activity in patients with colorectal cancer, primary and acquired treatment resistance is common. Identification of molecular markers at either the intra-genic, chromosomal, mRNA or protein level that might predict whether colorectal cancer patients are likely to benefit from systemic therapy is a subject of intense investigation.

Prognostic markers provide information about the probable course of the disease in individual patients and the chances of remaining progression- or disease-free or surviving independent of the therapy received. Predictive markers reflect the chance of benefit from a specific therapeutic intervention. A variety of retrospective studies have been performed over the past decade to identify molecular markers that may correlate with metastatic colorectal cancer response to 5-FU-based therapy. Expression of individual genes at the mRNA level can be determined using real-time reverse transcription, polymerase chain reaction (RT-PCR). Immunohistochemistry (IHC), enzyme-linked immunosorbent assays, and measurement of enzymatic activity may be employed to assess protein content. Analysis of genetic polymorphisms is a more recent approach.

Many of these retrospective studies in advanced colorectal cancer used various schedules of 5-FU modulated by leucovorin. With the availability of new agents with different mechanisms of action, and second- and third-line salvage therapies, the question of whether a given molecular marker will retain prognostic and/or predictive value is a subject of ongoing investigation.

5-FU exerts cytotoxicity by both RNA- and DNA-directed mechanisms. With the use of leucovorin and continuous infusion schedules, the contribution of thymidylate synthase (TS) inhibition is thought to be of primary importance. Inhibition of TS by 5-fluoro-2'-deoxyuridine monophosphate leads to imbalance of deoxyribonucleotide triphosphate pools (depletion of thymidine triphosphate, accumulation of deoxyuridine triphosphate and deoxyadenosine triphosphate), interference with DNA synthesis and repair, and induction of DNA strand breaks [2]. A variety of pre-clinical studies have demonstrated that over-expression of TS is associated with insensitivity to 5-FU. Several clinical studies that analyzed TS protein or mRNA expression in metastatic tumor tissue support the observation that low TS expression predicts for response [3-11].

Thymidine phosphorylase (TP) catalyzes the reversible conversion of thymidine to thymine and 5-fluoro-2'-deoxyuridine to 5-FU, a process that is influenced by the availability of 2'-deoxyribose-1-phosphate [12]. TP, also known as platelet-derived endothelial growth factor, appears to play a role in angiogenesis. High intratumoral TP expression has been associated with insensitivity to 5-FU therapy in some pre-clinical and clinical studies [13-15]. Capecitabine is an oral 5-FU pro-drug that is converted to 5-FU through a series of three enzymatic steps; intracellular TP mediates the final conversion of 5'-doxy-5-fluorouridine (doxifluridine) to 5-FU. Over-expression of TP in preclinical models is associated with increased sensitivity to capecitabine or doxifluridine, due to greater conversion to 5-FU [16,17]. TP is over-expressed in many human tumors compared to adjacent normal tissue, providing a potential for selective formation of 5-FU in tumor tissue [18]. Several studies have reported a correlation between elevated TP (as measured by either IHC or ELISA) and benefit with doxifluridine therapy [19-21].

Dihydropyrimidine dehydrogenase (DPD) catalyzes the rate-limiting step in the catabolism of 5-FU. Several clinical studies suggest that over-expression of DPD in colorectal tumor tissue is associated with poor response to 5-FU-based therapy [15,22-24].

Mammalian excision repair cross complementing protein (ERCC1) is involved in the repair of damaged DNA [25-27], and acts in concert with xeroderma pigmentosum protein F (XPF, also known as ERCC4 protein), which stabilizes ERCC1. This polypeptide complex introduces an incision on the 5' side of a damaged site in DNA. The ERCC1/XPF endonuclease complex is implicated in the repair of two distinct types of lesions in DNA: nucleotide excision-repair (NER), which is involved in repair of ultraviolet-induced lesions and bulky chemical adducts, and recombination repair of inter-strand cross-links. Over-expression of ERCC1 has been reported in cisplatin-resistant cancer cell lines [27]. Clinical data suggests that high intratumoral levels of ERCC1 mRNA are associated with insensitivity to oxaliplatin-based therapy in colorectal cancer [10].

The main purpose of this study was to determine a recommended dose of Xel given in combination with a fixed dose of OX on an every 3-week schedule. Correlations between gene expression of four molecular targets in tumor tissue and clinical outcome was a secondary endpoint.

## Methods

Subjects were participating in a Phase I study examining the feasibility of administering a fixed dose of oxaliplatin with escalating doses of capecitabine to patients with advanced colorectal or small bowel adenocarcinoma. The protocol was approved by the Cancer Therapy Evaluation Program, NCI, and Institutional Review Boards of the NCI and the National Naval Medical Center. All patients gave written informed consent.

Oxaliplatin 130 mg/m^2 ^was infused by vein over 2-hours on day one, while a total daily dose of capecitabine ranging from 1,200 to 3,000 mg/m^2 ^was given in two divided doses on days 1–5 and 8–12 of a planned 21-day cycle. Therapy was continued until evidence of disease progression occurred, unacceptable toxicity developed, or the patient elected to withdraw. Assessment of disease status was planned every three cycles.

Biopsies of metastatic tumor were obtained in 81 patients. Samples were placed in RNALater^® ^(Ambion Inc., Austin, TX, USA), stored at 4°C overnight, then stored at -30°C. Archival primary tumor tissue was available as formalin fixed, paraffin embedded (PFFE) tissue blocks in 27% of patients.

The clinical protocol document prospectively identified the four molecular targets of interest that would be tested, and specified that the median, first and third quartile values for relative gene expression for each of the four genes for all informative samples would be correlated with clinical outcome to see if there were any associations with greater or lesser mRNA expression.

### Micro-dissection

The frozen samples that had been stored in RNALater^® ^were embedded in optimal cutting temperature (OCT) compound (Secure Fanatic U.S.A., Inc., Torrance, CA) and cut into serial sections with a thickness of 20 am. Sections were mounted on unbolted glass slides and stored at -80°C. For histology diagnosis, three representative sections, consisting of the beginning, middle and end of sectioning were stained with hematoxylon and eosin (H&E) by the standard method.

Before micro-dissection, sections were air-dried, fixed in 70% ethanol for 3 min and washed in water for 2 min. Afterwards, they were stained with nuclear fast red (NER, American MasterTech Scientific, Inc., Lodi, CA, USA) for 10 sec and again washed in water for 30 sec. Samples were then dehydrated in a stepwise manner with 70% ethanol, 95% ethanol and 100% ethanol for 30 sec each, followed by incubation in xylene for 5 min and complete air-drying. All H&E stained sections were evaluated by a pathologist. If the histology of the samples was homogeneous and contained more than 90% tumor tissue, samples were dissected from the slides using a scalpel. All other sections were selectively isolated by LCM (P.A.L.M. Microsystem, Leica, Wetzlar, Germany) according to the standard procedure [28]. The micro-dissected tissue was transferred to a reaction tube containing 400 μL of RNA lysis buffer.

### RNA extraction

For extraction from FFPE specimens, the tissue samples to be extracted were placed in a 0.5 mL, thin walled tube containing 400 μL of 4 M dithiothreitol (DTT)-GITC/sarc (4 M guanidinium isothiocyanate, 50 mM Tris-HCl, pH 7.5, 25 mM EDTA) (Invitrogen; #15577-018). The samples were heated at 92°C for 30 min and then transferred to a 2 mL centrifuge tube. To the tissue suspensions were added 50 μL of 2 M sodium acetate, pH 4.0, followed by 600 μL of freshly prepared phenol/chloroform/isoamyl alcohol (250:50:1). The tubes were vortex-mixed for 15 sec, placed on ice for 15 min and then centrifuged at 13,000 rpm for 8 min in a chilled (8°C) centrifuge. The upper aqueous phase (250–350 μL) was carefully removed and placed in a 1.5 mL centrifuge tube. Glycogen (10 μL) and 300–400 μL of isopropanol were added and the samples vortex-mixed for 10–15 sec. The tubes were placed at -20°C for 30–45 min to precipitate the RNA. The samples were then centrifuged at 13,000 rpm for 7 min in a chilled (8°C) centrifuge. The supernatant was poured off and 500 μL of 75% ethanol was added. The tubes were centrifuged at 13,000 rpm for 6 min in a chilled (8°C) centrifuge. The supernatant was carefully poured off so as not to disturb the RNA pellet and the samples were quick-spun for 15 sec at 13,000 rpm. The remaining ethanol was removed with a 20 μL pipette and the samples air-dried for 15 min. The pellet was re-suspended in 50 μL of 5 mM Tris. For RNA extraction from OCT embedded samples, the above procedure was followed, except that the step involving heating at 92°C for 30 min was omitted.

### cDNA Preparation and Real Time PCR Quantification of mRNA Expression

cDNA was prepared as previously described [29]. Quantitation of TS, TP, ERCC1 and DPD and an internal reference gene (β-actin) was done using a fluorescence based real-time detection method (ABI PRISM 7900 Sequence detection System, Perkin-Elmer (PE) Applied Biosystem, Foster City, CA, USA). The PCR reaction mixture consisted 1200 nM of each primer, 200 nM probe, 0.4 U of AmpliTaq Gold Polymerase, 200 nM each dATP, dCTP, dGTP, dTTP, 3.5 mM MgCl_2 _and 1× Taqman^® ^Buffer A containing a reference dye, to a final volume of 20 μl (all reagents from PE Applied Biosystems, Foster City, CA, USA). Cycling conditions were 50°C for 2 min, 95°C for 10 min, followed by 46 cycles at 95°C for 15 sec and 60°C for 1 min. The primers and probes used are shown in Table 1.

**Table 1:** Sequences of primers and probes

Target	Forward Primer	Reverse Primer
TS	GCCTCGGTGTGCCCTTTCA	CCCGTTGATGTGCGCAAT

TP	CCTGCGGACGGAATCCT	TCCACGAGTTTCTTACTGAGAATGG

DPD	AGGACGCAAGGAGGGTTTG	GTCCGCCGAGTCCTTACTGA

ERCC1	GGGAATTTGGCGACGTAATTC	GCGGAGGCTGAGGAACGA

β-actin	TGAGCGGGCTACAGCTT	TCCTTAATGTCACGCACGATTT

Target	Probe: 6-FAM-5', 3'-TAMRA	

TS	TCGCCAGCTACGCCCTGCTCA	

TP	CAGCCAGAGATGTGACAGCCACCG	

DPD	CAGTGCCTACAGTCTCGAGTCTGCCAGTG	

ERCC1	CACAGGTGCTCTGGCCCAGCACATA	

β-actin	ACCACCACGGCCGAGCGG	

The cycle after which the fluorescent signal exceeds the threshold is referred to as the Ct. The Ct is inversely proportional to the amount of cDNA in the tube, i.e., a higher Ct value means that more PCR cycles are required to reach a certain level of detection. Gene expression values (relative mRNA levels) are expressed as ratios (differences between the Ct values) between the genes of interest and an internal reference gene (β-actin) that provides a normalization factor for the amount of RNA isolated from a specimen. Each cDNA sample had a duplicate genomic sample analyzed by real-time PCR without reverse transcription. If a fluorescent signal above the threshold was seen in the no reverse transcription specimen, the results from the corresponding cDNA were not deemed reliable. Each plate contained a reference cDNA sample to allow normalization for potential plate to plate variability.

Validation of the assay was performed with regard to RNA extraction and recovery, accuracy, precision, limits of quantitation and range. These studies followed GLP guidelines and employed Stratagene reference human RNA mix, pooled tissue homogenates derived from FFPE tissue sections and multiple sections of FFPE tumor sections from a variety of tumors. Assay precision was inferred from the variation in the replicate Ct values for all genes used, and had no more than 5.3% coefficient of variation (CV). The Delta Ct values derived from these Ct values showed a maximum %CV of 9.5%. This was expected, since the Delta Ct values reflect the variation in the Ct values of two genes, the target gene and its actin reference gene.

### Statistical and Graphical Analysis

Statistical analyses were performed using SigmaStat for Windows Version 3.11 (Systat Software Inc., Richmond, CA, USA). The median, first and third quartile values for each molecular marker were determined using all informative samples. The distribution of response vs. stable disease vs. early progressive disease as a function of molecular marker expression was analyzed by the chi-square test, whereas differences in response plus stable disease vs. early progressive disease was by Fisher's exact test. The strength of association of two variables was determined by the Pearson product moment correlation. Analysis of progression-free survival was performed by log-rank. Overall survival was analyzed by the Gehan-Breslow method. Graphs were prepared using SigmaPlot for Windows version 9.0 (Systat Software, Inc., Chicago, IL).

## Results

### Patient Characteristics and Clinical Outcome

Ninety-one subjects participated in this clinical trial (Table 2). One patient withdrew after having a tumor biopsy performed, and did not receive any protocol therapy. Six additional patients are not assessable for response. Among 84 subjects who were considered assessable for response to protocol therapy, there were four complete and 14 partial responses (21%). Forty-five patients had stable disease (54%), while 21 (25%) experienced clinical or radiographic evidence of disease progression by the time of initial restaging after 3 cycles. For all 91 subjects, the median time to treatment failure was 5.1 months, and the median survival was 10.9 months.

**Table 2:** Patient demographics (n = 91)

Median Age (Range)	56 yr (26–74)
Performance Status (ECOG)	
0	45
1	40
2	6

Male/Female	61/30

Primary Tumor Site	
colon	69
rectum	19
appendix	2
jejunum	1

Number Prior Chemotherapy Regimens	

0	4
1	40
2	37
3	10

Prior 5-fluoropyrimidine	87
Prior Irinotecan	73
Prior Radiation	19

### Tumor Tissue

Biopsies of metastatic tumor tissue were obtained in 81 subjects. Fifteen of these samples were not informative for any of the four targets of interest, either due to absence of tumor in the sample, or an insufficient quantity of tissue. The number of samples that were informative for each target was as follows: TS, 65; DPD, 63; TP, 64; ERCC1, 64. Archival paraffin-embedded formalin fixed tissue blocks from the primary tumor were available in 25 subjects. No tumor was seen in one of these. One additional subject had a biopsy of a rectal primary that was stored in RNALater^®^. The median time from initial diagnosis to study entry in subjects who had both archival primary tumor tissue and metastatic tumor tissue was 671 days (25^th^%, 426; 75^th^%, 824).

Table 3 shows the results of the gene expression for the pre-selected molecular targets. There was much greater variability in the distribution of values for each molecular target in metastatic tissue: the ratio of the maximum to minimum values ranged from 24 to 95. In primary tumor tissue, in contrast, the ratio of maximum to minimum values for each target ranged from 9 to 14.

**Table 3:** Relative gene expression in tumor tissue (vs. β-actin)

Molecular Marker	TS	DPD	TP	ERCC1
Metastasis (No.)	65	63	64	64

Median (range)	3.91 (0.81–77.58)	1.62 (0.15–11.89)	5.64 (1.06–31.89)	2.17 (0.34–8.23)

25^th^%, 75^th^%	2.38, 6.31	0.92, 3.04	3.77, 10.34	1.48, 3.36

Primary (No.)	24	21	24	25

Median (range)	2.07 (0.5–4.49)	0.36 (0.15–1.41)	3.23 (0.71–10.02)	1.04 (0.34–3.28)

25^th^%, 75^th^%	1.26, 2.58	0.21, 0.55	1.54, 4.98	0.64, 1.55

**Figure 1:**
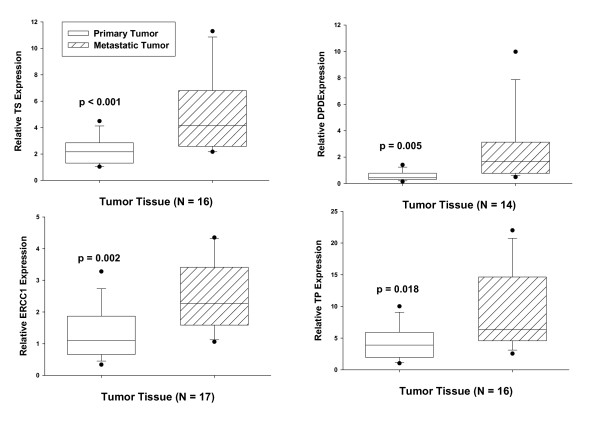
**Gene expression in paired primary and metastatic tumor tissue samples**. The data are shown in box-plot format; all outliers are shown. The p values are from a Wilcoxon signed rank test.

When mRNA expression was compared in paired samples of primary and metastatic tumor tissue, gene expression was significantly higher in metastatic tumor tissue for each molecular marker: TS, 1.9-fold; DPD, 3.8-fold; TP, 1.6-fold; ERCC1, 2.1-fold (Figure 1).

**Figure 2:**
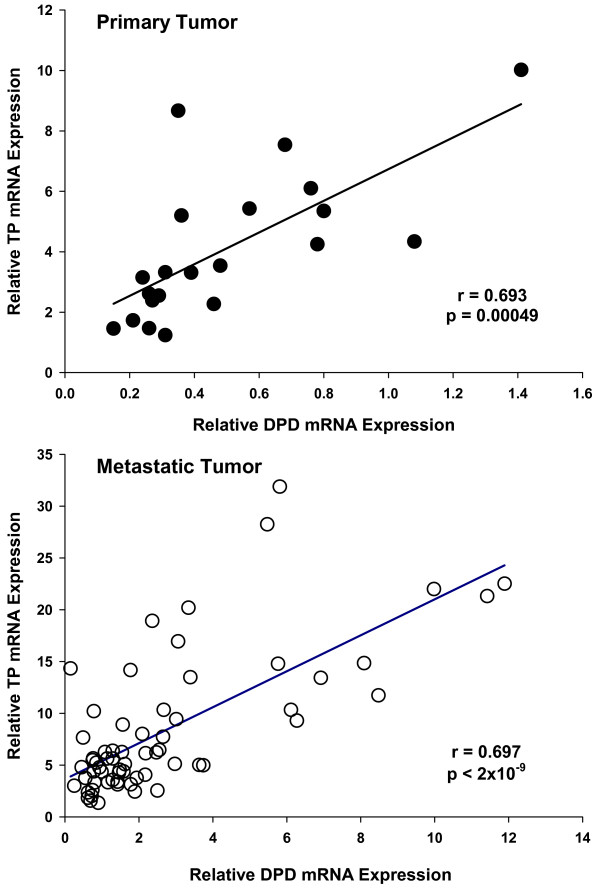
**Correlation between DPD and TP gene expression in either primary or metastatic tumor tissue**. The Pearson product moment correlation coefficient and p-value are shown.

The strength of association between doublets of molecular markers was analyzed. In both primary and metastatic tumor tissue, there was a strong correlation between the expression of DPD and TP (Figure 2). No other significant correlations were observed.

Restaging computerized tomographic scans were planned after the first three cycles (nine weeks). Individual target gene expressions were stratified as above or below either the median, first or third quartile. The proportion of observations in the different categories that defined the contingency tables were analyzed by comparing complete and partial response (CR and PR) versus stable disease (SD) versus early progressive disease (PD, within the first 9 weeks), or CR plus PR plus SD versus early PD. The only association that appeared to be non-random was with TS (Table 4). Patients whose intra-tumor TS mRNA levels were above the median value had significantly greater risk of early disease progression (43% vs 17%). Subjects who had very high intra-tumor ERCC1 expression tended to have an increased risk of early PD (47% vs 22%).

**Table 4:** Gene expression and disease control with XelOX therapy

	CR+PR+SD	PD	total
TS ≤ median	25	5	30

TS > median	17	13	30

ERCC1 ≤ 75^th^%	35	10	45

ERCC1 > 75^th^%	8	7	15

Six subjects were not assessable for response; therefore, only 60 subjects each with informative TS and ERCC1 samples from metastatic tumor tissue are included in the analysis between gene expression and disease control. The Fisher exact test p-value was 0.047 for TS and 0.099 for ERCC1.

**Figure 3:**
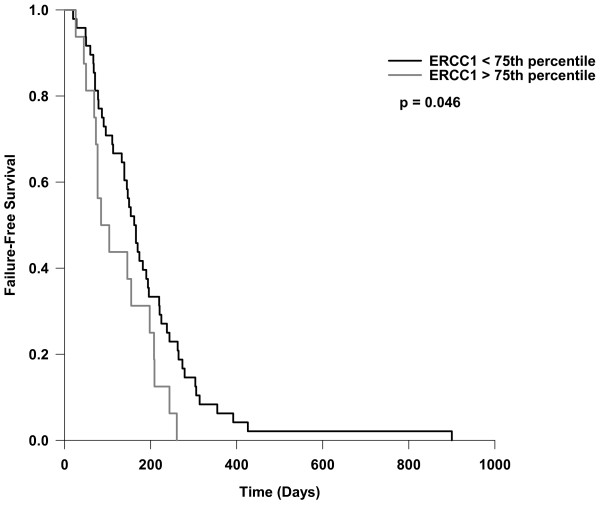
**Time to treatment failure as a function of ERCC1 gene expression**. The median TTF was 162 days in those with ERCC1 expression < 3^rd ^quartile (n = 46), compared to 85 days in those with expression > 3^rd ^quartile (n = 16). The log-rank p-value is shown.

Because a major endpoint of this study was tolerability of the regimen, a primary endpoint was time to treatment failure, which was defined as the interval from enrollment on study to either documented disease progression or the date a patient withdrew from the study for unacceptable toxicity. Only two patients withdrew from study due to unacceptable toxicity accompanied by a decline in performance status. Neither patient had a restaging CT scan, so the date of progression cannot be determined. When individual molecular marker expression was analyzed as a possible predictive factor for TTF, those subjects whose metastatic tumor tissue had ERCC1 gene expression above the third quartile had a significantly shorter TTF of 77 days (Figure 3). If the two patients who withdrew for toxicity are excluded, the result did not appreciably change: those with ERCC1 gene expression above the third quartile was significantly shorter: median TTF 85 days vs 166 days (p = 0.017). Various combinations of molecular markers did not discriminate any apparent differences in TTF.

**Figure 4:**
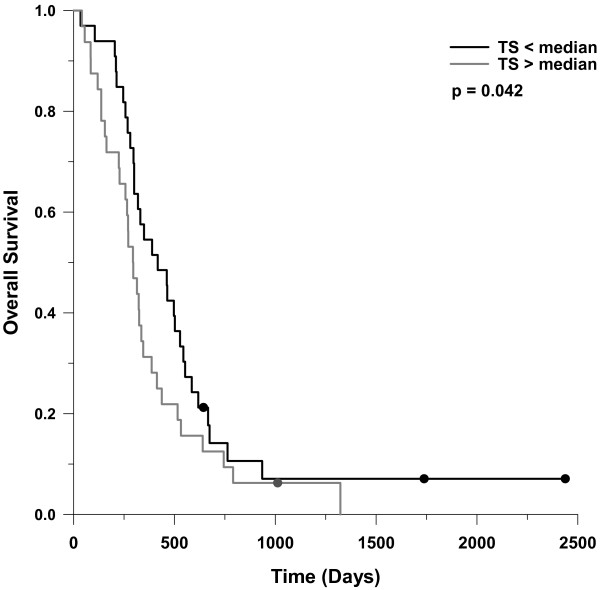
**Survival from on-study date as a function of TS gene expression**. The median survival was 417 days in those with TS expression < median (n = 33), compared to 294 days in those with expression > median (n = 32). The Gehan-Breslow p value is shown.

Overall survival from the on-study date was analyzed according to higher versus lower mRNA expression of individual targets. The only individual molecular marker that tended to distinguish those with worse or better survival was TS (Figure 4). The median survival in subjects whose metastatic tumor tissue had TS mRNA expression below the median was 123 days longer than those with TS expression above the median. Various combinations of molecular marker expression were analyzed (e.g. TS below median and ERCC1 below third quartile vs others), but none yielded stronger prognostic information than TS mRNA expression alone.

## Discussion

The objective response rate was 21% in this heterogenous patient population with a median of two prior chemotherapy regimens. mRNA expression of TS, DPD, ERCC1 and ERCC1 in paired metastatic and primary tumor was significantly higher in metastatic tissue. This may in part be explained by the long interval from initial diagnosis to study entry (median 671 days). It is acknowledged that mRNA from primary tissue was derived from formalin fixed paraffin-embedded samples while metastatic tissues were immediately placed in RNA later and frozen. Frozen tissues typically generate more RT-PCR product than FFPE tissues [30]. However, normalization of gene expression to β-actin gene expression in the same tissue should correct for any relative differences in efficiency of RT-PCR product.

In both metastatic and primary tissue, there was a significant correlation between DPD and TP mRNA expression. The genes encoding DPD and TP are located on chromosomes 1p22 and 22q13, respectively. Other investigators have also reported a significant correlation between mRNA levels of DPD and TP in both primary and metastatic tumor tissue [31,32]. Further studies would be needed to see if these genes are coordinately regulated.

Due to the small sample size and heterogeneity of the patient population, the results and conclusions of this analysis must be interpreted with caution. It appeared that TS mRNA expression below or above the median tended to distinguish those subjects who achieved either objective response or stable disease vs. those who had early disease progression within 9 weeks of enrolling on protocol. Subjects whose metastatic tumor tissue had ERCC1 mRNA expression greater than the 3rd quartile had a shorter median time to treatment failure (12 vs 23 weeks). It is assumed that higher TS and ERCC1 gene expression translates into greater protein expression and enzymatic activity.

The lack of importance of TS and TP gene expression in predicting TTF may possibly be explained by the trial design which involved dose escalation of Xel; some subjects received lower Xel doses than that eventually recommended. In this cohort, we observed that high DPD mRNA levels accompanied high TP mRNA levels. It is possible that the theoretical benefit of high TP expression, which is expected to lead to increased intracellular release of 5-FU, is offset by increased catabolism of 5-FU.

In summary, in this trial involving predominantly pre-treated colorectal cancer patients, higher levels of TS mRNA expression appeared to have a predictive value in identifying those subjects with a greater probability of early disease progression during treatment with XelOX. Subjects whose metastatic tumor tissue had TS mRNA expression below the median tended to have longer median survival by about 18 weeks. Since TS mRNA expression did not correlate with improved TTF, low TS expression in this study may be more of a prognostic than a predictive factor. Information on subsequent therapies is not available. The importance of gene expression of pertinent 5-FU-critical targets may be offset with combination therapy and the availability of salvage regimens. For example, high TS tumor expression does not preclude response to 5-FU given in combination with irinotecan [33,34].

Finally, there has been some discordance in reports concerning expression of various tumor markers in paired primary and metastatic disease. Our observation that the expression of these four tumor markers in metastatic tissue was significantly higher than the primary tumor in the setting of a relatively long interval between obtaining the specimens (1.8 years) highlights the importance of specifying the interval between procurement of these tumor samples in published manuscripts.

## Conclusion

Gene expression of TS, DPD, ERCC1 and ERCC1 in metastatic tissue was significantly higher than in primary tumor. High ERCC1 mRNA levels in metastatic tumor was associated with a shorter TTF. Lower expression of TS mRNA correlated with a lower chance of early PD with XelOX therapy and improved overall survival.

## Competing interests

Kazumi Uchida has no financial or non-financial competing interests to declare. 

Peter V. Danenberg has the following competing financial interests to declare: Holds shares of Response Genetics, Inc. There are no non-financial interests to declare.

Kathleen D. Danenberg has the following competing interests to declare: Receives salary from Response Genetics Inc. and holds shares of Response Genetics, Inc. There are no non-financial interests to declare.

Jean L. Grem has no financial or non-financial competing interests to declare.

## Authors' contributions

KU carried out the analysis of the tumor tissue, including embedding the samples in OTC compound, microdissection, RNA extraction, cDNA preparation, and real-time PCR quantitation. PVD and KDD analyzed the results of the real-time quantitative PCR. JLG was the principal investigator of the clinical trial, performed the correlations of the gene expression data with various clinical endpoints, and wrote the manuscript. All authors read and approved the final manuscript.

## Pre-publication history

The pre-publication history for this paper can be accessed here:


